# Beyond clinical outcomes: the social and healthcare system implications of hepatitis C treatment

**DOI:** 10.1186/s12879-020-05426-4

**Published:** 2020-09-24

**Authors:** Marta Torrens, Tokunbo Soyemi, Darcy Bowman, Eberhard Schatz

**Affiliations:** 1 Addiction Research Group, Hospital del Mar Medical Research Institute, Barcelona, Spain; 2 Department of Psychiatry and Forensic Medicine, Universitat Autònoma de Barcelona, Barcelona, Spain; 3 Medical Affairs, Gilead Sciences Ltd, London, UK; 4 Public Affairs, Gilead Sciences Ltd, London, UK; 5 Correlation European Harm Reduction Network, Amsterdam, The Netherlands

**Keywords:** Hepatitis C, Outcomes, Experience, People who inject drugs, Mental health, Physical wellbeing

## Abstract

**Background:**

Hepatitis C virus (HCV) infections in people who inject drugs (PWID) can now be treated and cured. However, the impact that HCV treatment has on drug-user health, practices and wellbeing is not known. The aim of this research was to understand the non-clinical impact that HCV treatment has in PWID and their reasons for accessing and completing treatment.

**Methods:**

Participants aged 25–67 years who had injected opioids or stimulants (currently or in the past) and had completed direct-acting antiviral treatment were recruited from seven European countries. Participants completed a 30-min online survey administered face-to-face between September 2018 and April 2019. The questionnaire responses were used to assess the mental and physical impact of having completed treatment.

**Results:**

Of the 124 participants who completed the survey questionnaire, 75% were male, 69% were over 45 years old and 65% were using opioids and/or stimulants at the start of HCV treatment. Participants reported improvements in the following areas after completing HCV treatment: outlook for the future (79%); self-esteem (73%); ability to plan for the future (69%); belief in their abilities (68%); confidence (67%); empowerment (62%); energy levels (59%); and ability to look after themselves (58%). The most common reasons for starting HCV treatment were: becoming aware of treatments that were well tolerated (77%) and effective (75%); and understanding the potentially severe consequences of HCV (75%).

**Conclusions:**

The benefits of HCV treatment go beyond clinical outcomes and are linked to improved drug-user health and wellbeing. Sharing information about well-tolerated and effective HCV treatments, and raising awareness about the potentially severe consequences of untreated HCV are likely to increase the number of PWID who are motivated to access and complete HCV treatment in future.

## Background

Chronic hepatitis C is an important public health problem, with an estimated 71 million people living with the hepatitis C virus (HCV) across the globe in 2015 [1]. If left untreated, hepatitis C can progress to serious liver disease; the estimated probability of cirrhosis at 20 years after HCV infection is 16% [2].

The recent introduction of simple, highly effective and well-tolerated direct-acting antiviral (DAA) treatment regimens for HCV provides an opportunity to reduce prevalence and ultimately eliminate HCV infection as a public health threat [3]. For example, significant progress towards HCV elimination has been made in Iceland, with 81% (603/741) of people identified as HCV-positive cured through a 3-year national elimination programme [4]. To achieve HCV elimination, treatment of populations at high risk of infection such as people who inject drugs (PWID), should be prioritised [5]. In Europe, HCV prevalence among people with recent injection drug use is between 39.9 and 48.6% whereas HCV prevalence in the general population is approximately 1.5% [1, 6]. In fact, injection drug use is the main driver of HCV infection in Europe, with approximately 80% of new cases attributable to this high-risk behaviour [7]. Although DAA therapy can cure the majority of people living with HCV, there are still some physical and psychological barriers that prevent people, including PWID, from accessing HCV care and treatment [8, 9]. The psychological impact of living with hepatitis C is well-documented with many people experiencing depression and anxiety [10, 11]. These symptoms, which are very common among PWID, may affect people’s willingness to initiate HCV treatment [12, 13].

There is a gap in knowledge on the factors that encourage PWID to start DAA treatment and on the impact treatment has on drug-user health, practices and wellbeing. Therefore, the aim of this investigation was to survey injection drug users who have received DAA treatment in order to gain insights on the non-clinical impact that HCV treatment has had on their life, and on wider society. Additionally, the study aimed to ascertain the main reasons these participants were motivated to access and complete HCV treatment, as well as survey the participants’ opinions on the factors that may encourage more PWID to engage in HCV care.

## Methods

### Study population

Participants included in this study were aged between 25 and 67 years, were either injecting opioids or stimulants regularly (at least once a week), injecting occasionally (less than once a week) or had previously injected opioids or stimulants. The participants had also received a confirmed diagnosis of hepatitis C (self-reported) and completed DAA treatment. Participants were incentivised to take part in the study in the form of vouchers. Each participant was offered physical or digital vouchers (supermarket or Amazon) worth 50 euros (£50 in the UK).

### Recruitment

The aim was to recruit eight injection drug users per country in the UK (Brighton and Blackpool), France (Montpellier and Marseille), Germany (Essen and Cologne), Italy (Rome) and Spain (Bilbao), and six injection drug users per country in Portugal (Lisbon and Porto) and Switzerland (Lugano). Recruitment channels in these countries included patient associations/organisations, referrals from healthcare professionals (HCPs), recruiter panels (local databases of patients and HCPs from a variety of specialties), patient referrals and online campaigns. Invitations to participate in the survey were sent via these channels in September 2018. A team of local fieldwork partners/recruiters with experience in healthcare market research conducted recruitment in each of the countries. Physicians (from the recruiter panels) and patient associations/organisations were contacted by email or telephone to invite them to assist with patient referrals. In this communication, they were provided with a letter detailing information about the study, invited to help refer suitable patients and given details of the referral incentive. The incentive consisted of donations ranging from 100 to 1000 euros or £100–£1000 in the UK (depending on how many participants were referred) to charities and addiction centres for their support in referring participants. Physical or digital vouchers (supermarket or Amazon) worth 50 euros (£50 in the UK) were offered to participants who referred other participants to the survey. In Portugal, there were no incentives given for referrals due to country regulations. No healthcare professionals were paid any referral incentives. Physicians were invited to tell patients about the study and provide them with the recruiter’s contact details. Patients were free to contact the recruiter if they were interested in participating. The recruiters also advertised the study and provided information about how to take part via various channels including the recruiter panel website, social media and patient association forums, newsletters and websites.

### Survey

The survey questionnaire was developed by Incite, an independent international market research consultancy (Supplementary File 1). The authors, who have a wide knowledge of hepatology and addiction, contributed to the development of the questionnaire and decisions on which outcomes to measure. They reviewed the final version to ensure alignment with the research objectives and that the questions were appropriate for participants. The survey comprised of 25 questions relating to participants’ sociodemographic characteristics, injecting behaviours and HCV treatment. The survey also included seven questions on the perceived impact of HCV treatment on different aspects of the participants’ lives. The survey was hosted online on the Confirmit platform.

Once participants had agreed to participate, the online survey was conducted in-person (as a face-to-face interview) in the native language at a convenient location (e.g. a café, HCV nurse’s office, rehabilitation centre, drug addiction clinic or patient’s home). The interviews were carried out by moderators (non-HCPs) with experience working in healthcare market research, including patient research. All moderators had received training on how to ask questions on topics of a sensitive nature. At the beginning of every interview, participants were reassured on the anonymity of their responses to encourage open and honest feedback. All responses provided by the participant were inputted onto a tablet or laptop by the moderator. Data were collected on the Confirmit platform. Incite researchers and analysts collated and analysed the data using Q Research Software.

### Data collection

The surveys were carried out from 13 September 2018 to 11 April 2019 and took 30 min to complete on average.

## Results

### Baseline characteristics of participants

In this study, 124 participants completed the survey questionnaire. The target for the minimum number of participants per country was met with 100 respondents from the UK, Germany, France, Italy and Spain (20 per country), 15 from Portugal and 9 from Switzerland. Of the 124 participants who completed the survey, the majority (75%) were male and aged over 44 years old (69%). Many received a confirmed HCV diagnosis before 2008 (73%) and concurrent illnesses were common (Table 1). Overall, 65% of participants were using opioids and/or stimulants at the start of HCV treatment, and the other 35% had a history of injection drug use. Most participants were recruited through a patient association/organisation (62%) or through a referral from an HCP (20%). The majority (78%) received treatment less than 3 years ago and treatment was most often received from a secondary care hospital (73%).

**Table 1:** Baseline characteristics of survey participants (*N* = 124)

Characteristics	%
Age, years	
18–29	2
30–44	30
45–60	64
> 60	5
Sex	
Male	75
Female	25
Year HCV diagnosis was received	
Before 1999	40
1999–2003	15
2004–2008	18
2009–2013	13
2014–2018	15
Comorbidities (more than one could be selected)	
HBV	13
HIV	24
Cardiovascular disease	7
Diabetes	2
Other	13
Currently injecting drugs	
≥ once a week	17
< once a week	17
No longer injecting	66
Use of opioids/stimulants at start of HCV treatment	
Users	65
Non-users	34
Did not remember	1
Substances used	
Opioids (e.g. heroin, morphine)	92
Stimulants (e.g. cocaine, amphetatmine)	85
Both opioids and stimulants	78
Other substances	58
Country	
UK	16
Germany	16
France	16
Italy	16
Spain	16
Portugal	12
Switzerland	7
Recruitment channel	
Patient association/organisation	62
HCP referral	20
Recruiter panel	13
Patient referral	3
Online campaign	2
Year started most recent HCV treatment	
≥ 5 years ago	8
4 years ago	5
3 years ago	10
2 years ago	19
1 year ago	33
This year	26
Where most recent HCV treatment was received	
Secondary care/hospital	73
Addiction clinic	14
GP/PCP	5
Prison	2
Other	3
HCV treatment most recently received^a^ (more than one could be selected)	
SOF/VEL	27
LDV/SOF	23
SOF	14
GLE/PIB	6
DCV	5
EBR/GZR	2
TEL	2
SOF/VEL/VOX	2
OBV/PTV/r	2
DSV	2
BOC	2
Other/unknown	23

^a^The treatment regimen received by participants was at the discretion of their treating physician and was a function of the timing of the research, and/or regulatory approvals and reimbursement in each country

*BOC* Boceprevir, *DCV* Daclatasvir, *DSV* Dasabuvir, *EBR* Elbasvir, *GLE* Glecaprevir, *GP* General practitioner, *GZR* Grazoprevir, *HBV* Hepatitis B virus, *HCP* Healthcare professional, *HCV* Hepatitis C virus, *HIV* Human immodeficiency virus, *LDV* Ledipasvir, *OBV* Ombitasvir, *PCP* Primary care physician, *PIB* Pibrentasvir, *PTV* Paritaprevir, *r* Ritonavir, *SOF* Sofosbuvir, *TEL* Telaprevir, *VEL* Velpatasvir, *VOX* Voxilaprevir

### Self-perceived impact of HCV treatment on mental health and physical wellbeing

**Fig. 1:**
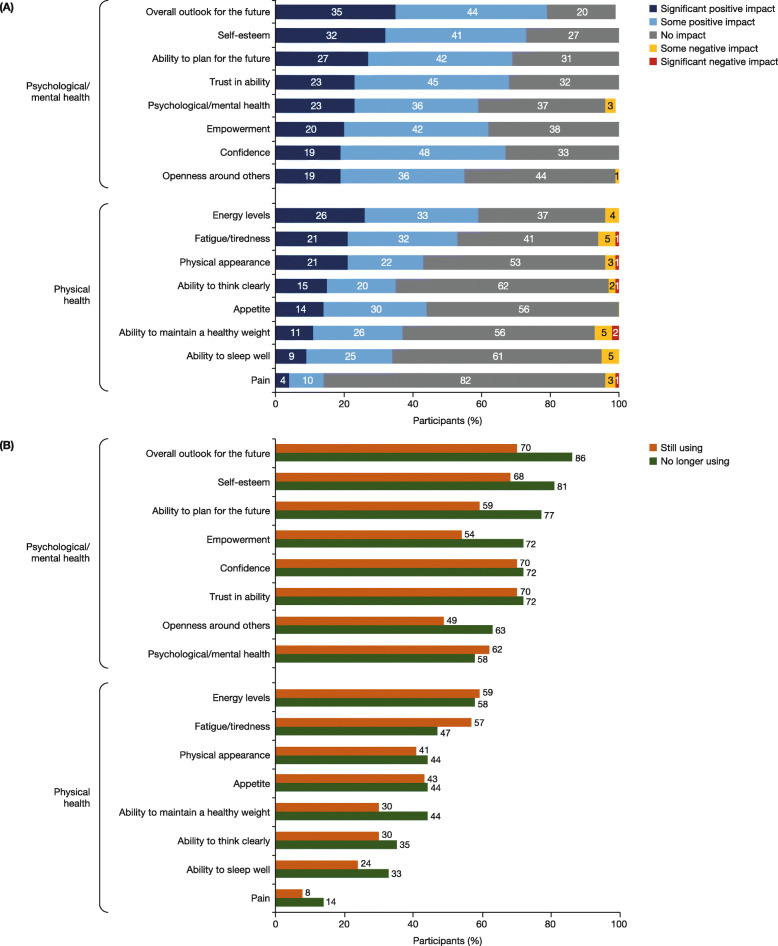
**a** The impact of HCV treatment on participants’ mental health and physical wellbeing (*N* = 124). **b** The positive impact (defined as either a significant positive impact or some positive impact) of HCV treatment on mental health and physical wellbeing among participants who used opioids/stimulants at the start of treatment (*n* = 80). Those who no longer used opioids/stimulants at the time of the survey (n = 43, green) were compared with those who still did (n = 37, orange)

Participants reported on the impact (significant positive, some positive, some negative, significant negative or none) of HCV treatment on various mental and emotional treatment-related outcomes (Fig. 1a). A positive impact (defined as either a significant positive impact or some positive impact) with HCV treatment was reported by a high proportion of participants on the following outcomes: outlook for the future (79%); self-esteem (73%); ability to plan for the future (69%); belief in their abilities (68%); and confidence (67%). A small number of participants reported a negative impact (defined as either some negative impact or significant negative impact) on psychological health (3%) and their ability to be open with others (1%). For all mental health-related outcomes except psychological/mental health, the percentage of people who reported a positive impact with HCV treatment was higher in participants who no longer used opioids/stimulants at the time of the survey compared with those who did (Fig. 1b).

For physical health, a positive impact on energy levels and fatigue/tiredness with HCV treatment was reported in 59 and 53% of participants, respectively (Fig. 1a). Few participants reported a negative impact on their physical wellbeing, with the most common negative outcomes being tiredness/fatigue (6%) or inability to maintain a healthy weight (6%). Additionally, most participants (82%) reported that HCV treatment had no impact on pain. Among participants who used opioids/stimulants at the start of therapy, the parameter with the greatest numerical difference between participants who no longer used opioids/stimulants at the time of the survey and those who did was the ability to maintain a healthy weight (44% compared with 30%) (Fig. 1b).

### Self-perceived impact of HCV treatment on lifestyle, ability to engage in wider society and ability to maintain personal relationships

**Fig. 2:**
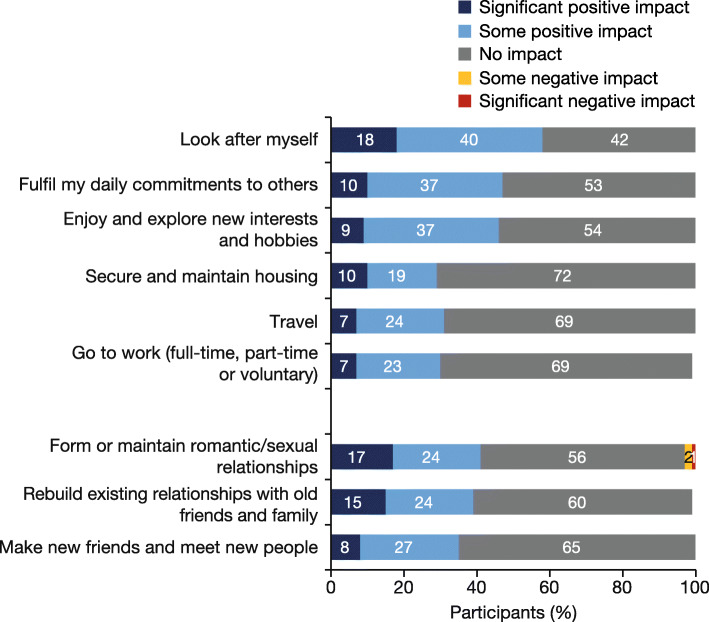
The impact of HCV treatment on the ability of participants to maintain personal relationships and aspects of their lifestyle (*N* = 124)

Over half (58%) of participants reported a positive impact in their ability to look after themselves and just under half (47%) reported a positive impact on their ability to fulfil daily commitments to others (Fig. 2). Overall, 46% reported a positive impact on their ability to enjoy/explore new interests and hobbies with many participants starting new activities after completing treatment: 6% started a sport or hobby; 13% obtained a new job; 19% enrolled in an education or training course; and 30% became an advocate regarding HCV and/or drug use (e.g. advising patients, companies or healthcare professionals) (Supplementary Fig. 1). For the parameters related to maintaining personal relationships, 35–41% of participants reported a positive impact with HCV treatment; the majority reported no impact.

### Accommodation and employment status before HCV treatment and during survey

There were few changes to the participants’ accommodation before and after HCV treatment: living in their own home from 36 to 44%, respectively; in a family home from 22 to 25%; in shared accommodation from 18 to 13%; in a halfway house from 10 to 9%; homeless from 5 to 2%; rehabilitation/detox centre from 5 to 4%; and in prison from 2 to 0% (Supplementary Fig. 2). Employment status remained largely the same before and after completing HCV treatment: on benefits from 35 to 31%, respectively; out of work from 44 to 41%; employed (full- or part-time) from 24 to 23%; retired from 8 to 7%; and volunteer/peer work from 6 to 5% (Supplementary Fig. 3).

### Self-reported emotions felt after completing treatment

**Fig. 3:**
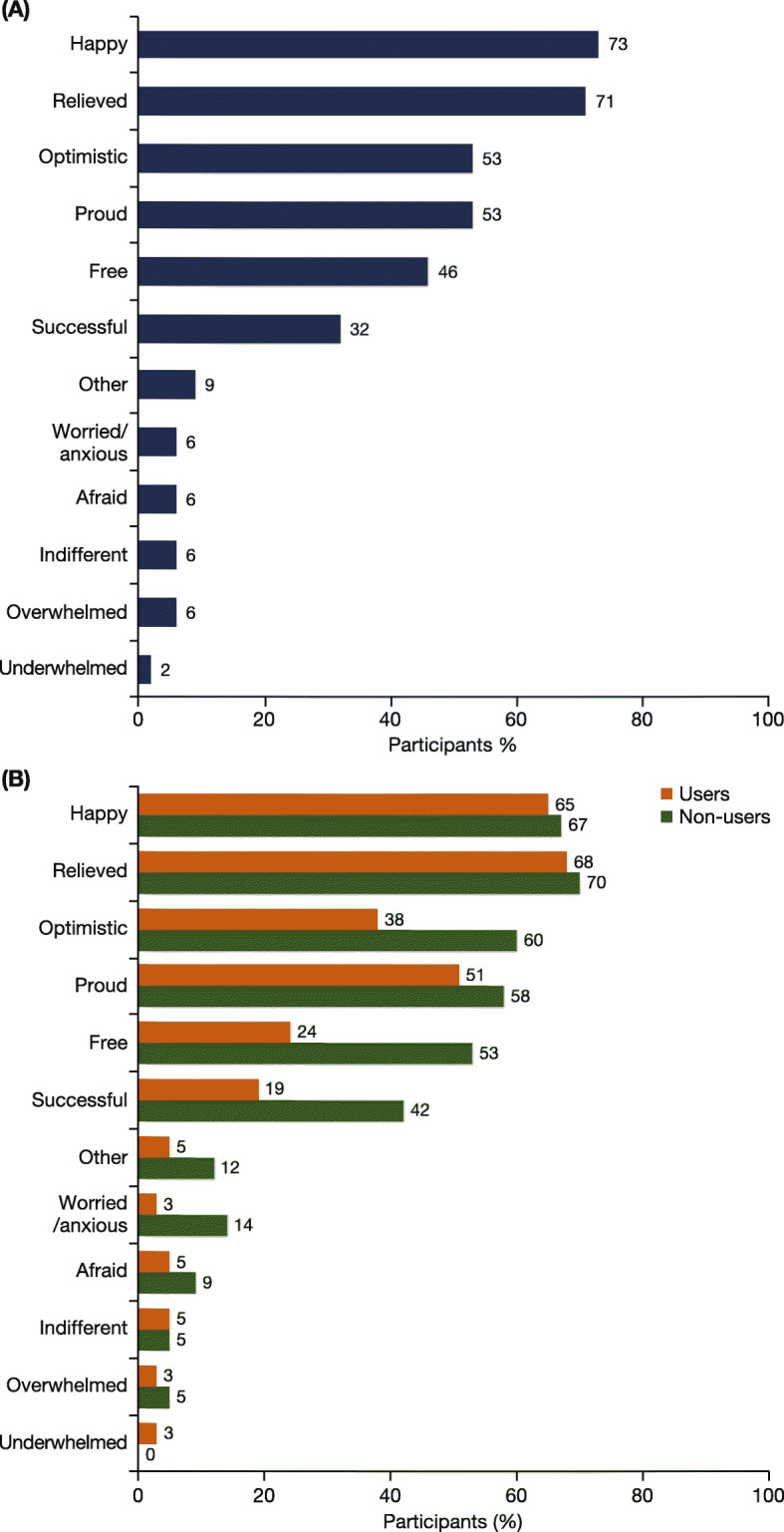
**a** Emotions felt by participants after completing HCV treatment (N = 124). Participants were able to select more than one emotion. **b** Emotions felt by participants who used opioids/stimulants at the start of treatment (*n* = 80). Those who no longer used opioids/stimulants at the time of the survey (*n* = 43, green) were compared with those who still did (*n* = 37, orange)

The most common emotions reported by participants after completing treatment were: happy (73%), relieved (71%), optimistic (53%); and proud (53%) (Fig. 3a). Among participants who used opioids/stimulants at the start of therapy, the most common emotions felt after treatment were: relieved (70% in participants who no longer used drugs at the time of the survey, 68% in users at the time of survey) and happy (67% in non-users, 65% in users) (Fig. 3b). The emotions with the greatest numerical difference between participants who no longer used opioids/stimulants at the time of the survey and those who did were: successful (42% in non-users, 19% in users), free (53% in non-users, 24% in users) and optimistic (60% in non-users, 38% in users).

### Motivations for starting HCV therapy

**Fig. 4:**
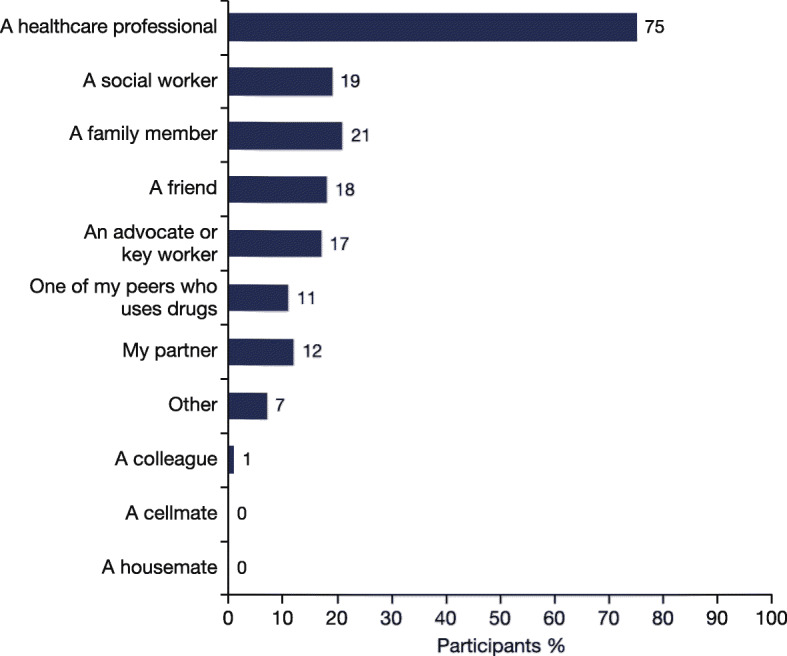
The person who encouraged each participant to start HCV treatment (*N* = 84). Participants were able to select more than one person

During the survey, 84 participants reported that someone else encouraged them to start treatment. Of those 84 participants: 75% were encouraged by an HCP; 21% by a family member; 19% by a social worker; 18% by a friend; 17% by an advocate or key worker; 12% by their partner; and 11% by one of their peers (Fig. 4).

**Fig. 5:**
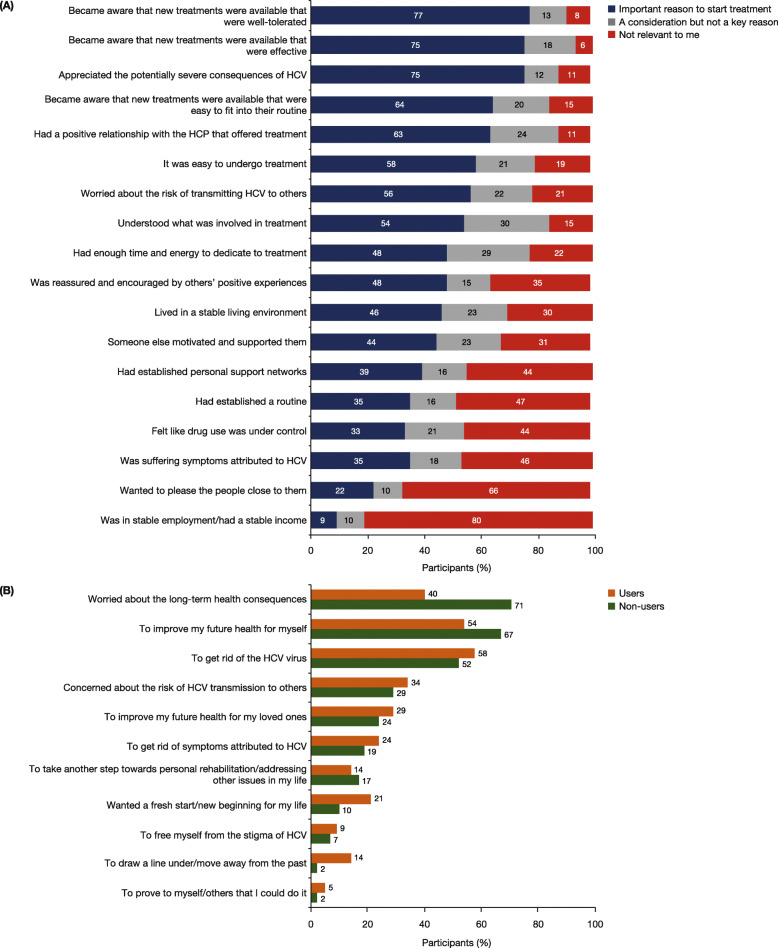
**a** Reasons for participants to start treatment (N = 124). **b** Top three motivations for starting HCV treatment among participants using opioids/stimulants at the start of their treatment (n = 80, orange) and non-using participants (*n* = 42, green). Participants were asked to rank their top three motivations (ranking 1 to 3), those who provided rankings for all three of their top motivations were included in the analysis

**Fig. 6:**
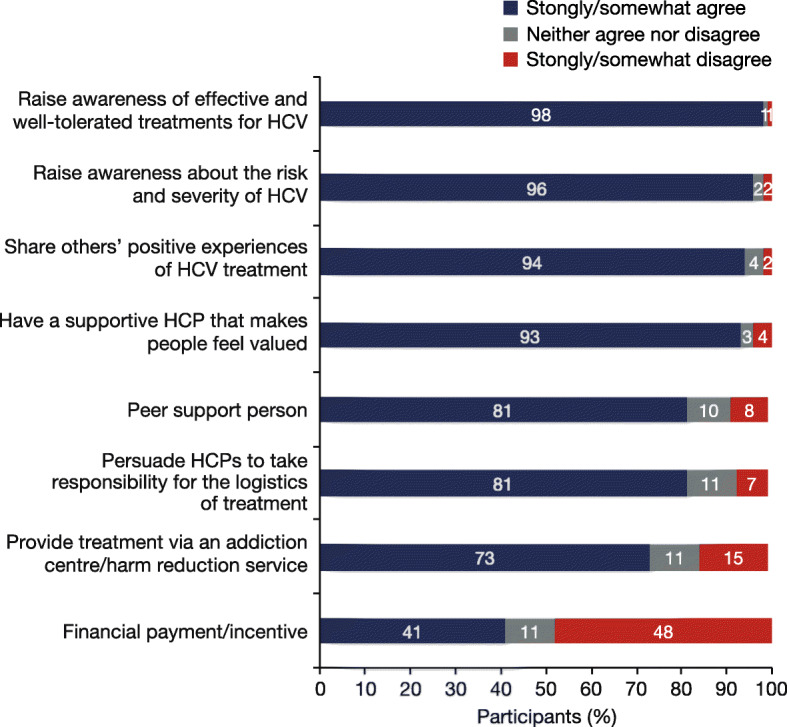
Potential initiatives that participants agreed would help other PWID to start and complete HCV treatment (N = 124)

The majority of participants (≥75%) stated that becoming aware of treatments that were effective and well tolerated, as well as understanding the potentially severe consequences of HCV, were their main reasons for starting therapy (Fig. 5a). Only 35% of individuals identified that suffering symptoms of HCV was a motivation to start treatment. In the sub-population of individuals not using opioids/stimulants at the start of HCV treatment, 71% were concerned about the long-term consequences of the disease (Fig. 5b)**.** For those who were drug users at the start of HCV treatment, the most frequently reported reason to start therapy was ‘to get rid of the virus’. The participants agreed that raising awareness of effective and well-tolerated treatments (98%), raising awareness about the risk and severity of HCV (96%), hearing about the positive experiences of others (94%) and having a supportive HCP (93%) were the factors that were most likely to encourage others to start and complete HCV treatment (Fig. 6). The factor that was the least likely to encourage others was financial payments/incentives (41%).

## Discussion

This survey analysis shows that there are additional benefits to HCV treatment in PWID other than cure; many participants experienced a positive impact on other outcomes, mainly in their subjective psychological/mental health. These results align with previous studies which demonstrate that HCV treatment has a transformative effect on patients’ quality of life and mental wellbeing [14–17].

Despite these benefits, global HCV treatment uptake among PWID is low [18, 19]. There are many system-level and provider-level reasons for this low treatment uptake but at an individual level, lack of awareness of HCV diagnosis, previous negative experiences with healthcare systems, stigma and physical/psychological co-morbidities have been identified as common barriers for PWID [20–22]. Another key barrier is lack of knowledge or misinformation about HCV and how it is treated [20]. Until 2013, the only treatments that were available for HCV were interferon (IFN)-based and required weekly injections for up to 1 year [23]. These IFN-based regimens were associated with many side effects and low efficacy rates, particularly among PWID [24, 25]. Negative experiences of these poorly tolerated regimens have been shared among drug user communities which has resulted in many people being fearful of treatment side effects [26, 27].

With the advent of well-tolerated and orally administered DAA drugs, HCV treatment has become simpler and the majority of infected individuals, including PWID, can be treated and cured [28]. There is now consensus among the medical community that all patients should be able to access care and that treating PWID in particular is a priority to reduce HCV transmission [5, 29]. Pangenotypic regimens that are currently available require minimal pre-, during and post-treatment monitoring and can be used effectively to treat large numbers of previously underserved populations, thus maximising efforts towards HCV elimination [30].

There is still a need to understand what motivates PWID to engage in care. Qualitative research has illustrated that eliminating the virus is not the only factor that motivates PWID to start therapy [31]. The results of our survey suggest that awareness of the availability of effective and well-tolerated treatments and the potentially severe consequences of untreated HCV are key motivators. This highlights the importance of developing programmes and initiatives that include patient education to ensure that more PWID are treated for HCV [32]. In the Netherlands, education and awareness campaigns targeted at PWID have already been proven to be successful at engaging more individuals into care, with 257 additional PWID diagnosed with chronic HCV when these interventions were used compared to the control group [33]. An interesting topic for further research would be to survey PWID who decline HCV treatment to understand which factors influence their decision making.

Even in the era of simple DAA treatments that are effective in many patient populations, management of HCV among PWID is often perceived as challenging because this population is usually stigmatised by society and can find healthcare services difficult to access [21, 22, 34]. However, findings from this survey suggest that the majority of PWID reported a positive impact on their ability to engage in wider society and explore new interests/hobbies after HCV treatment. Previous studies have also shown similar positive outcomes beyond HCV cure that are valued highly among PWID [16, 31].

In this analysis, there was wide variation in injecting patterns between participants, with different numbers of participants injecting drugs before and after HCV treatment. This highlights the complexity of this patient population and should be considered when interpreting the results. For example, for some outcomes such as overall outlook for the future, ability to plan for the future and empowerment, there was a large difference in the number of participants who reported a positive impact with HCV treatment between those who used drugs at the time of the survey and those who did not. It is not clear why this is the case however it suggests that injection drug use could also have an impact on these outcomes. Previous research has demonstrated that stopping drug use can improve an individual’s mental and physical health, and therefore their quality of life [35, 36].

The results of this research should be interpreted in light of several limitations. First, given the nature of the data, it was not appropriate to conduct statistical analyses so we cannot draw reliable statistical comparisons between different categories of patients. Therefore, our results should be viewed as indicators for future studies. The cross-sectional nature of the survey must also be taken into account when analysing these results as for many participants, there was variation in the time between completing treatment and completing the survey. In addition, this study only presents data on the aspects of life mentioned in the survey and so there may be more benefits of treatment that are not described. Finally, we report results from a survey of PWID who had volunteered and were therefore motivated to participate. We cannot guarantee that the findings are representative of all people who use drugs.

It is also important to highlight the specific strengths of this research. For example, this is one of the first analyses that focus on the self-perceived impact of HCV treatment on mental health and physical wellbeing in people who inject drugs. Many of the outcomes that we report on have not been measured in clinical trials and so these findings add breadth to the literature on patient-level benefits of HCV treatment. In addition, information from this survey allows us to gain insights on a high-risk and highly stigmatised population that is usually excluded from healthcare programmes and large randomised controlled clinical trials. One of the benefits of this study was that recruitment of participants was driven by patients with the majority recruited through patient associations/organisations. A possible reason for this is that PWID visit community-based organisations more regularly than other healthcare settings [37]. In general, this survey shows that having a variety of different recruitment channels is valuable when recruiting PWID.

## Conclusions

In this survey analysis, we show that the benefits of HCV treatment among PWID go beyond clinical outcomes and are linked to improved health and wellbeing. In addition, our results suggest that awareness of effective and well-tolerated HCV treatments, as well as understanding the potentially severe consequences of untreated HCV are the main reasons PWID are motivated to access and complete HCV treatment.

## Supplementary information

**Additional file 1: Supplementary File 1**. Survey Questionnaire

**Additional file 2: Supplementary Figure 1**. Impact of HCV treatment on participants’ ability to engage in wider society (*N* = 124). **Supplementary Figure 2**. Participants’ living situation at the start of HCV treatment and during the survey (N = 124). **Supplementary Figure 3**. Participants’ employment status at the start of HCV treatment and during the survey (N = 124).

## Data Availability

The datasets used and/or analysed during the current study are available from the corresponding author on reasonable request.

## Abbreviations

DAA: Direct-acting antiviral
HCP: Healthcare professional
HCV: Hepatitis C virus
IFN: Interferon
PWID: People who inject drugs
