# Supplementary material for: Beyond clinical outcomes: the social and healthcare system implications of hepatitis C treatment
Source: BMC Infect Dis. 2020 Sep 24;20:702. doi: 10.1186/s12879-020-05426-4 (PMC7517680; doi:10.1186/s12879-020-05426-4)
Supplement: Supplementary file 1 — Additional file 1: Supplementary File 1. Survey Questionnaire [file 12879_2020_5426_MOESM1_ESM.pdf]

**Supplementary File 1. Survey Questionnaire**

## *Screening questions*

S1/ Do you have a history of substance use?

Please select all that apply.

| | |
|---|---|
| Yes, I have used opioids like heroin or morphine | |
| Yes, I have used stimulants like cocaine, amphetamine, methamphetamine | |
| Yes, I have used other substances | |
| No, I have not | |

S2/ What methods have you ever used to take these substances?

Please select all that apply.

| | Opioids like heroin or morphine | Stimulants like cocaine, amphetamine, methamphetamine |
|---|---|---|
| Smoking | | |
| Snorting | | |
| Injecting | | |

S3/ Which of the following best describes your current situation with regards to your use of injectable opioids and/or stimulants?

| | |
|---|---|
| I am still injecting opioids/stimulants regularly | |
| I am still injecting opioids/stimulants occasionally | |
| I am no longer injecting opioids/stimulants but have done so in the past | |

S4/ Which, if any, of the following conditions have you been diagnosed with by a doctor?

Please select all that apply.

| | |
|---|---|
| Cardiovascular disease | |
| Diabetes | |
| Hepatitis C | |
| Hepatitis B | |
| HIV | |
| Other | |

S5/ When did you receive a confirmed diagnosis for hepatitis C (i.e. when did you first receive confirmed hepatitis C blood test results)?

S6/ Since, you were diagnosed with hepatitis C, have you received treatment?

| | |
|---|---|
| Yes, I am currently receiving treatment for hepatitis C | |
| Yes, I have received and completed treatment for hepatitis C | |
| No, I have not received treatment for hepatitis C | |

S7/ Which treatment have you received **most recently** for hepatitis C?

| | |
|---|---|
| Boceprevir (Victrelis) | |
| Daclatasvir (Daklinza) | |
| Dasabuvir (Exviera) | |
| Elbasvir/grazoprevir (Zepatier) | |
| Glecaprevir/pibrentasvir (Maviret) | |
| Interferon-based treatment | |
| Ledipasvir/sofosbuvir (Harvoni) | |
| Ombitasvir/paritaprevir/ritonavir (Viekirax) | |
| Simeprevir (Olysio) | |
| Sofosbuvir (Sovaldi) | |
| Sofosbuvir/velpatasvir (Epclusa) | |
| Sofosbuvir/velpatasvir/voxilaprevir (Vosevi) | |
| Telaprevir (Incivo) | |
| Other (please specify) | |
| None of the above | |

S8/ And when was it that you started treatment for hepatitis C?

S9/ And how long did you take HCV treatment for?

***Questions relating to experience with hepatitis C treatment***

Q1/ In the month prior to starting your **most recent** treatment for hepatitis C, how often were you using opioids and/or stimulants?

| | Less than once per month | Every few weeks | Weekly | Daily | Multiple times per day | I don't remember | I was not using opioids/ stimulants |
|---|---|---|---|---|---|---|---|
| Opioids like heroin or morphine | | | | | | | |
| Stimulants like cocaine, amphetamine, methamphetamine | | | | | | | |

Q2/ In the month prior to starting your **most recent** treatment for hepatitis C, which of the following best describes your living situation?

*I lived in…*

| | |
|---|---|
| My family home | |
| My own flat/home | |
| A shared flat/home | |
| A halfway house or hostel | |
| A rehabilitation/detox centre | |
| Prison | |
| Homeless | |
| Other (please specify) | |

Q3/ In the month prior to starting your **most recent** treatment for hepatitis C, which of the following best describes your employment status?

| | |
|---|---|
| Employed full time | |
| Employed part time | |
| Out of work but looking for work | |
| Out of work but not looking for work | |
| Volunteer/peer work | |
| A home maker (e.g. housewife or househusband) | |
| A student | |
| Retired | |
| On benefits (e.g. disability benefit) | |
| Prefer not to say | |

Q4/ In the month prior to starting your **most recent** treatment for hepatitis C, were you engaged with any healthcare professionals regarding your use of opioids and/or stimulants?

*These healthcare professionals could be a GP, psychiatrist, addiction specialist, harm reduction worker, nurse, counsellor, psychologist, key worker or social worker based in, for example, your local doctor's surgery, drug services or addiction centre, mental health services or needle exchange services.*

| | |
|---|---|
| Yes | |
| No | |

*Q5/* In the month prior to starting your **most recent** treatment for hepatitis C, which of the following describes your main goal with regards to your use of opioids and/or stimulants?

| | |
|---|---|
| I wanted to stop using opioids and/or stimulants | |
| I wanted to reduce my use of opioids and/or stimulants | |
| I wanted to make an increased effort to minimise the risks associated with using opioids and/or stimulants | |
| I did not want to change my use of opioids and/or stimulants | |

*Q6/* Which service offered you your **most recent** treatment for hepatitis C?

| | |
|---|---|
| **UK** | |
| Drug and alcohol service via hepatologist in-reach | |
| Community pharmacy | |
| Secondary care / hospital setting | |
| Prison | |
| Primary care physician / GP | |
| **Harm reduction services** | |
| Drug consumption rooms | |
| Needle and syringe programmes | |
| Opioid substitution therapy | |
| Other community setting (please specify) | |
| Other (please specify) | |
| **FR** | |
| CSAPA (Centres de soins, d'Accompagnement et de prevention en addictologie) | |

| | |
|---|---|
| CAARUD (Les Centres d'Accueil et d'Accompagnement à la Réduction des risques pour Usagers de Drogues) | |
| CSAPA/CAARUD | |
| Mobile clinic | |
| **Harm reduction services** | |
| Drug consumption rooms | |
| Needle and syringe programmes | |
| Opioid substitution therapy | |
| Secondary care / hospital setting | |
| Prison | |
| Primary care physician / GP | |
| Other community setting (please specify) | |
| Other (please specify) | |
| **DE** | |
| Addiction clinic | |
| Secondary care / hospital setting | |
| Prison | |
| Primary care physician / GP | |
| **Harm reduction services** | |
| Drug consumption rooms | |
| Needle and syringe programmes | |
| Opioid substitution therapy | |
| Other community setting (please specify) | |
| Other (please specify) | |
| **IT** | |
| SERT (Servizi Tossicodipendenze) | |
| Secondary care / hospital setting | |
| Prison | |
| Primary care physician / GP | |
| **Harm reduction services** | |
| Drug consumption rooms | |
| Needle and syringe programmes | |
| Opioid substitution therapy | |
| Other community setting (please specify) | |

| | |
|---|---|
| Other (please specify) | |
| **ES** | |
| Addiction clinic | |
| Secondary care / hospital setting | |
| Prison | |
| Primary care physician / GP | |
| **Harm reduction services** | |
| Drug consumption rooms | |
| Needle and syringe programmes | |
| Opioid substitution therapy | |
| Other community setting (please specify) | |
| Other (please specify) | |
| **PO** | |
| Addiction clinic | |
| **Harm reduction services** | |
| Drug consumption rooms | |
| Needle and syringe programmes | |
| Opioid substitution therapy | |
| Secondary care / hospital setting | |
| Prison | |
| Primary care physician / GP | |
| Other community setting (please specify) | |
| Other (please specify) | |
| **CH** | |
| HAT (Heroin Assisted Therapy) | |
| **Harm reduction services** | |
| Drug consumption rooms | |
| Needle and syringe programmes | |
| Opioid substitution therapy | |
| Secondary care / hospital setting | |
| Prison | |
| Primary care physician / GP | |
| Other community setting (please specify) | |
| Other (please specify) | |

*Q7/* Where did you receive your **most recent** treatment for hepatitis C?

| | |
|---|---|
| **UK** | |
| Drug and alcohol service via hepatologist in-reach | |
| Community pharmacy | |
| Secondary care / hospital setting | |
| Prison | |
| Primary care physician / GP | |
| **Harm reduction services** | |
| Drug consumption rooms | |
| Needle and syringe programmes | |
| Opioid substitution therapy | |
| Other community setting (please specify) | |
| Other (please specify) | |
| **FR** | |
| CSAPA (Centres de soins, d'Accompagnement et de prevention en addictologie) | |
| CAARUD (Les Centres d'Accueil et d'Accompagnement à la Réduction des risques pour Usagers de Drogues) | |
| CSAPA/CAARUD | |
| Mobile clinic | |
| **Harm reduction services** | |
| Drug consumption rooms | |
| Needle and syringe programmes | |
| Opioid substitution therapy | |
| Secondary care / hospital setting | |
| Prison | |
| Primary care physician / GP | |
| Other community setting (please specify) | |
| Other (please specify) | |
| **DE** | |
| Addiction clinic | |
| Secondary care / hospital setting | |
| Prison | |

| | |
|---|---|
| Primary care physician / GP | |
| **Harm reduction services** | |
| Drug consumption rooms | |
| Needle and syringe programmes | |
| Opioid substitution therapy | |
| Other community setting (please specify) | |
| Other (please specify) | |
| **IT** | |
| SERT (Servizi Tossicodipendenze) | |
| Secondary care / hospital setting | |
| Prison | |
| Primary care physician / GP | |
| **Harm reduction services** | |
| Drug consumption rooms | |
| Needle and syringe programmes | |
| Opioid substitution therapy | |
| Other community setting (please specify) | |
| Other (please specify) | |
| **ES** | |
| Addiction clinic | |
| Secondary care / hospital setting | |
| Prison | |
| Primary care physician / GP | |
| **Harm reduction services** | |
| Drug consumption rooms | |
| Needle and syringe programmes | |
| Opioid substitution therapy | |
| Other community setting (please specify) | |
| Other (please specify) | |
| **PO** | |
| Addiction clinic | |
| **Harm reduction services** | |
| Drug consumption rooms | |
| Needle and syringe programmes | |
| Opioid substitution therapy | |

| | |
|---|---|
| Secondary care / hospital setting | |
| Prison | |
| Primary care physician / GP | |
| Other community setting (please specify) | |
| Other (please specify) | |
| **CH** | |
| HAT (Heroin Assisted Therapy) | |
| **Harm reduction services** | |
| Drug consumption rooms | |
| Needle and syringe programmes | |
| Opioid substitution therapy | |
| Secondary care / hospital setting | |
| Prison | |
| Primary care physician / GP | |
| Other community setting (please specify) | |
| Other (please specify) | |

*Q8/* What made you start treatment for hepatitis C at this time (thinking about your **most recent** treatment)?

Please select all that apply.

| **Disease related reasons** | Important reason for me to start treatment | A consideration but not a key reason for me to start treatment | Not relevant to me |
|---|---|---|---|
| I appreciated the potentially severe consequences of hepatitis C | | | |
| I was suffering from symptoms that were attributed to hepatitis C | | | |
| I was worried about the risk of transmitting hepatitis C to others | | | |
| **Treatment related reasons** | | | |
| I became aware that new treatments were available that were effective (associated with cure/getting rid of the virus) | | | |
| I became aware that new treatments were available that were well tolerated (with minimal side effects) | | | |
| I became aware that new treatments were available that were easy to fit into my routine | | | |

| | | | |
|---|---|---|---|
| I understood what was involved with treatment | | | |
| It was easy to undergo treatment - minimal effort was required on my part | | | |
| **Encouragement from/by others** | | | |
| I was reassured and encouraged by others' positive experiences of hepatitis C treatment | | | |
| Someone else motivated me and supported me to start hepatitis C treatment | | | |
| I had a positive relationship with the healthcare professionals that offered me treatment | | | |
| I wanted to please people that were close to me | | | |
| **Personal situation had changed** | | | |
| I felt like I had my drug use under control | | | |
| I lived in a stable living environment | | | |
| I had established a routine | | | |
| I had established personal support networks | | | |
| I had enough time and energy to dedicate to treatment | | | |
| I was in stable employment/had a stable income | | | |

*Q9/* Who encouraged you to start your **most recent** hepatitis C treatment?

| | |
|---|---|
| One of my peers that uses drugs | |
| An advocate or key worker | |
| A social worker | |
| My partner | |
| A family member | |
| A friend | |
| A healthcare professional | |
| A colleague | |
| A cellmate | |
| A housemate | |
| Other (please specify) | |

*Q10/* What were your top three motivations for starting your **most recent** hepatitis C treatment?

Please rank your top three statements from the list below, where 1 would be the most motivating reason, 2 would be the second most motivating reason and 3 would be the third most motivating reason.

| | |
|---|---|
| I was worried about the long-term health consequences of hepatitis C | |
| I was always concerned about the risk of hepatitis C transmission to others | |
| I wanted to free myself from the stigma of hepatitis C | |
| I wanted to get rid of symptoms attributed to hepatitis C | |
| I wanted to get rid of the hepatitis C virus | |
| I wanted to draw a line under/move away from the past | |
| I wanted a fresh start/new beginning for my life | |
| I wanted to improve my future health for my loved ones (e.g. family, partners, friends) | |
| I wanted to improve my future health for myself | |
| I wanted to take another step towards personal rehabilitation/addressing other issues in my life | |
| I wanted to prove to myself and/or others that I could do it | |
| None of the above/I was not personally motivated to start treatment | |

Q11/ Which of the following is the main reason you continued and completed treatment for hepatitis C?

| | |
|---|---|
| I remained motivated by the outcome/benefit that the treatment would give me | |
| The treatment was easy to fit into my daily life | |
| The process of treatment was straightforward, e.g. taking the treatment itself | |
| The healthcare system/professionals made it easy for me to continue treatment, e.g. organised appointments, sent me reminders, regular contact | |
| I was encouraged by healthcare professionals | |
| I was encouraged by my partner, family, friends and/or peers | |
| None of the above | |

Q12/ Below is a list of statements showing some ideas that could encourage more people who have injected substances like opioids and/or stimulants to begin and complete treatment for hepatitis C based on some recent research in this area.

To what extent do you agree with the following statements?

| | Strongly agree | Somewhat agree | Neither agree nor disagree | Somewhat disagree | Strongly disagree |
|---|---|---|---|---|---|
| More education and understanding about the risk and severity of hepatitis C would motivate people to get tested or treated for hepatitis C earlier | | | | | |
| More awareness of available treatments for hepatitis C that are effective and safe would motivate people get tested or treated for hepatitis C earlier | | | | | |
| Hearing about positive experiences of hepatitis C treatment from friends or others who have been/are in a similar situation would encourage people to seek treatment earlier | | | | | |
| If healthcare professionals took responsibility for the logistics (e.g. organising appointments, monitoring treatment, sending reminders), then more people would start and continue hepatitis C treatment | | | | | |
| People are more likely to start and continue hepatitis C treatment, if it is offered, administered and monitored through an addiction centre/harm reduction service or other community-based service (as opposed to at hospital) | | | | | |
| People are more likely to start and continue hepatitis C treatment, if their health care professional is supportive and makes them feel valued | | | | | |
| People are more likely to start and continue hepatitis C treatment, if they have a peer support person to provide support throughout the process | | | | | |
| People are more likely to start and continue hepatitis C treatment, if they are paid/financially incentivised to do so | | | | | |

## *Questions related to the impact of hepatitis C treatment*

Q13/ Which of the following words best describe how you felt **emotionally** when you completed your **most recent** treatment for hepatitis C?

Please select all that apply.

| | |
|---|---|
| Relieved | |
| Free | |
| Happy | |
| Afraid | |
| Worried/anxious | |
| Optimistic | |
| Proud | |
| Indifferent | |
| Overwhelmed | |
| Underwhelmed | |
| Successful | |
| Other (please specify) | |

Q14/ Now that you have completed your **most recent** treatment for hepatitis C, how has this impacted the following aspects of your physical wellbeing?

| | Significant positive impact | Some positive impact | No impact | Some negative impact | Significant negative impact |
|---|---|---|---|---|---|
| Energy levels | | | | | |
| Fatigue/tiredness | | | | | |
| Appetite | | | | | |
| Ability to maintain a healthy weight | | | | | |
| Ability to sleep well | | | | | |
| Pain | | | | | |
| Physical appearance | | | | | |
| Ability to think clearly, e.g. concentration, memory | | | | | |
| Psychological/mental health | | | | | |

Q15/ Now that you have completed your **most recent** treatment for hepatitis C, how has this impacted the following aspects of your personal life?

| | Significant positive impact | Some positive impact | No impact | Some negative impact | Significant negative impact |
|---|---|---|---|---|---|
| **Your relationships** | | | | | |
| Ability to make new friends and meet new people | | | | | |
| Ability to rebuild existing relationships with old friends and family | | | | | |
| Ability to form or maintain romantic/sexual relationships | | | | | |
| **Your approach to the future** | | | | | |
| Your overall outlook for the future | | | | | |
| The extent to which you plan for the future | | | | | |
| **The way that you feel about yourself** | | | | | |
| Self-esteem | | | | | |
| Confidence | | | | | |
| Empowerment (feeling able to do something/take control) | | | | | |
| Trust in ability | | | | | |
| Openness to and with others | | | | | |

Q16/ Now that you have completed your **most recent** treatment for hepatitis C, how has this impacted the following aspects of your day-to-day life?

| | Significant positive impact | Some positive impact | No impact | Some negative impact | Significant negative impact |
|---|---|---|---|---|---|
| Ability to secure and maintain housing | | | | | |
| Ability to look after myself (e.g. basic self-care) | | | | | |
| Ability to fulfil my daily commitments to others (for example taking care of family or children) | | | | | |
| Ability to go to work (for example full or part-time work, voluntary work) | | | | | |

| | | | | | |
|---|---|---|---|---|---|
| Ability to enjoy and explore new interests and hobbies (for example playing sports/exercising, reading) | | | | | |
| Ability to travel (for example going on holiday, seeing friends/family) | | | | | |

Q17/ Have you started any of the following activities since completing your **most recent** treatment for hepatitis C?

Please select all that apply.

| | |
|---|---|
| I have enrolled in an education or a training course | |
| I have a new job/employment | |
| I have become an advocate regarding Hepatitis C and/or drug use, e.g. advising patients, companies or healthcare professionals | |
| Other (please specify) | |
| None of the above | |

Q18/ Since receiving your **most recent** treatment for hepatitis C, how, if at all, has your drug using behaviour changed?

| | |
|---|---|
| I have since stopped my use of opioids and/or stimulants | |
| (Follow up from statement above) I have remained abstinent from opioid and/or stimulant use | |
| I have since reduced my use of opioids and/or stimulants | |
| I have not changed my use of opioids and or stimulants | |
| I have since increased my use of opioids and/or stimulants | |

Q19/ Since receiving your **most recent** treatment for hepatitis C, which of the following best reflects how your approach to drug use behaviour has changed?

| | |
|---|---|
| Hepatitis C treatment and cure kick started my rehabilitation from opioid and/or stimulant use | |
| Hepatitis C treatment and cure gave me more determination and commitment to ongoing rehabilitation from opioid and/or stimulant use | |
| Since receiving hepatitis C treatment, I have made an increased effort to minimise the risks associated with using opioids and/or stimulants e.g. I make more of an effort to use clean, sterile injecting equipment, reduced sharing of equipment | |
| Hepatitis C treatment and cure has not changed my approach to opioid and/or stimulant use | |
| Hepatitis C treatment and cure has made me less committed or determined to reduce my opioid and/or stimulant use | |
| Hepatitis C treatment and cure has kick started an increase in my opioid and/or stimulant use | |
| Since receiving hepatitis C treatment, I have made less of an effort to minimise the risks associated with using opioids and/or stimulants e.g. I make less of an effort to use clean, sterile injecting equipment, I make less of an effort to reduce sharing of equipment | |

## *Classification questions*

C1/ How old are you?

C2/ Are you...?

| | |
|---|---|
| Male | |
| Female | |
| Transgender | |

C3/ Which of the following best describes your working status?

| | |
|---|---|
| Employed full time | |
| Employed part time | |
| Out of work and looking for work | |
| Out of work but not currently looking for work | |
| Volunteer/peer work | |
| A home maker | |
| A student | |

| | |
|---|---|
| Retired | |
| On benefits (e.g. disability benefit) | |
| Prefer not to say | |

C4/ Which of the following best describes your living situation? I live…

| | |
|---|---|
| In my family home | |
| In an independent flat/ house | |
| In a shared flat/ house | |
| At a halfway house or hostel | |
| In a rehab/ detox centre | |
| In prison | |
| Homeless | |
| Other (please specify) | |
