# Supplementary material for: Beyond clinical outcomes: the social and healthcare system implications of hepatitis C treatment
Source: BMC Infect Dis. 2020 Sep 24;20:702. doi: 10.1186/s12879-020-05426-4 (PMC7517680; doi:10.1186/s12879-020-05426-4)
Supplement: Supplementary file 2 — Additional file 2: Supplementary Figure 1. Impact of HCV treatment on participants’ ability to engage in wider society (N = 124). Supplementary Figure 2. Participants’ living situation at the start of HCV treatment and during the survey (N = 124). Supplementary Figure 3. Participants’ employment status at the start of HCV treatment and during the survey (N = 124). [file 12879_2020_5426_MOESM2_ESM.docx]

# Supplementary File 2. Supplementary figures


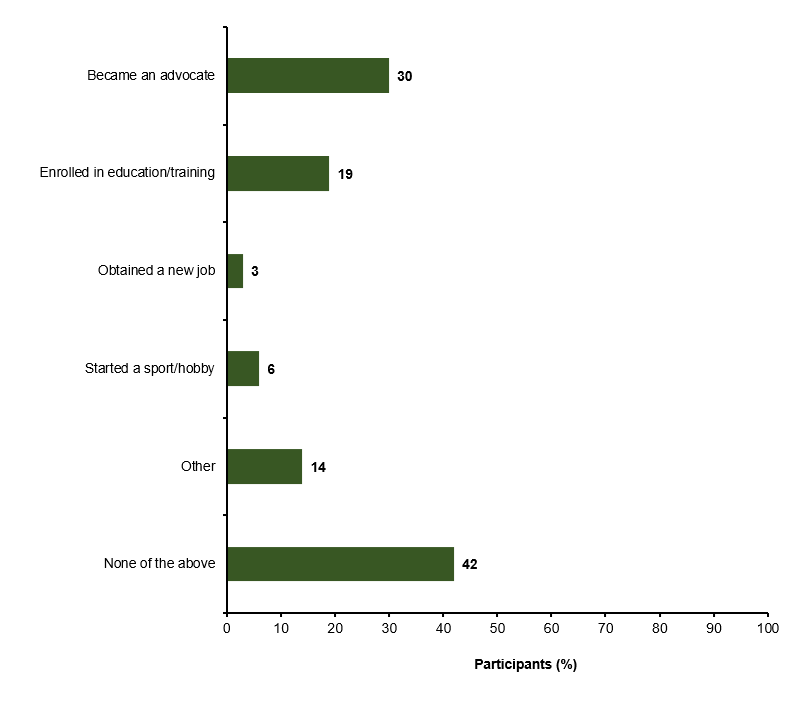
***Supplementary Figure 1. Impact of HCV treatment on participants' ability to engage in wider society (N=124)***

***Supplementary Figure 2. Participants’ living situation at the start of HCV treatment and during the survey (N=124)***


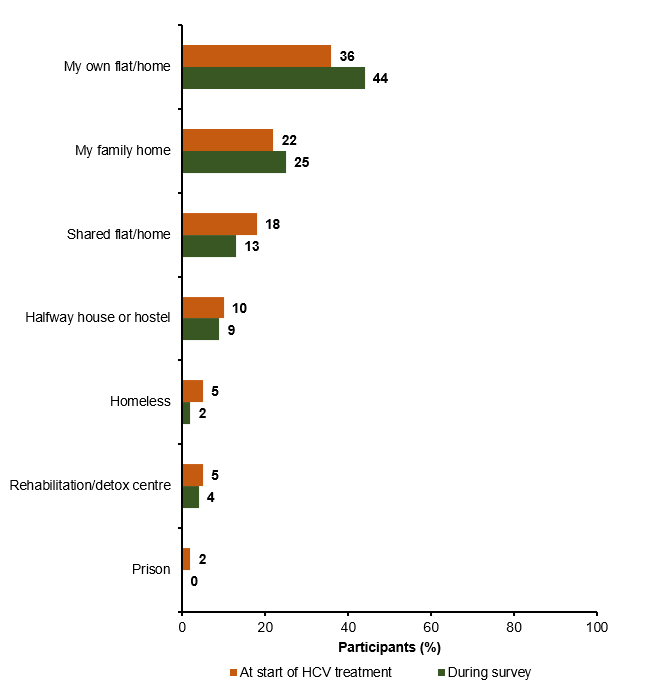


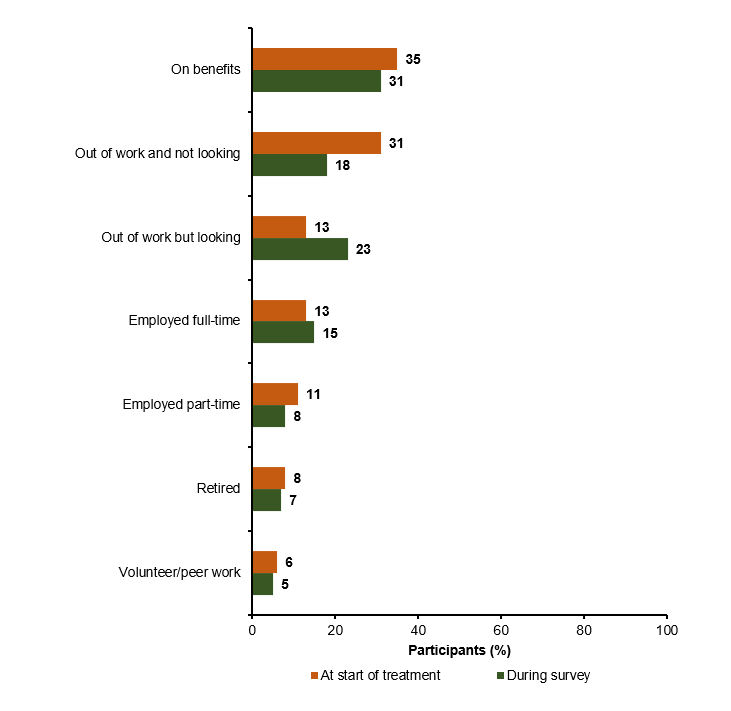
***Supplementary Figure 3. Participants’ employment status at the start of HCV treatment and during the survey (N=124)***
